# Acute myocardial infarction due to type A aortic dissection in a patient with corrected congenital cardiopathy: a case report

**DOI:** 10.1093/ehjcr/ytaf250

**Published:** 2025-05-22

**Authors:** G Padilla-Rodríguez, A Gómez-González, M Barquero-Alemán, I Méndez-Santos, J C García-Rubira

**Affiliations:** Cardiology Unit, Virgen Macarena Hospital, Avd. Dr. Fedriani 3, 41009 Seville, Spain; Cardiology Unit, Virgen Macarena Hospital, Avd. Dr. Fedriani 3, 41009 Seville, Spain; Cardiovascular Surgery, Virgen Macarena Hospital, Avd. Dr. Fedriani 3, 41009 Seville, Spain; Cardiac Imaging, Cardiology Unit, Virgen Macarena Hospital, Avd. Dr. Fedriani 3, 41009 Seville, Spain; Acute Cardiovascular Care Unit, Virgen Macarena Hospital, Avd. Dr. Fedriani 3, 41009 Seville, Spain

**Keywords:** acute myocardial infarction, Aneurysm of the ascending aorta, Aortic dissection, Case report

## Abstract

**Background:**

Ascending aortic dissection rarely presents as acute myocardial infarction, and when it does, its diagnosis is even more challenging.

**Case summary:**

We present a case of a young male with corrected congenital heart disease and a giant ascending aorta aneurysm. He was admitted to hospital for chest pain and was diagnosed with acute anterior myocardial infarction. Imaging tests showed dissection of the ascending aorta. He required complex cardiovascular surgery for Dacron tube implantation to treat the giant ascending aortic aneurysm.

**Discussion:**

This case is a reminder of how important adequate differential diagnosis is in the context of myocardial infarction, as well as the management of acute aortic syndrome. In our patient, the patency of the left coronary artery was verified using imaging techniques, and the most likely cause of the infarction could be the aneurysm extrinsically compressing the coronary artery.

**Learning points**

- A rare cause of coronary malperfusion in aneurysmal aortic dissection may be compression of the left main coronary artery. Aortic CT angiography is a fundamental diagnostic tool in these cases.
- Acute aortic syndrome is an entity that is difficult to diagnose and has a high mortality rate, which requires an emergent approach. Delaying surgery for aortic dissection in the presence of an extensive myocardial infarction should probably be an individual decision.

## Introduction

Ascending aortic dissection (AD) rarely presents as acute myocardial infarction (AMI), making its diagnosis particularly challenging. We present the case of a young man with corrected congenital heart disease and a giant ascending aortic aneurysm (AAA) who developed proximal AD and anterior myocardial infarction due to extrinsic compression of the left coronary artery (LCA).

## Summary figure

**Figure:**
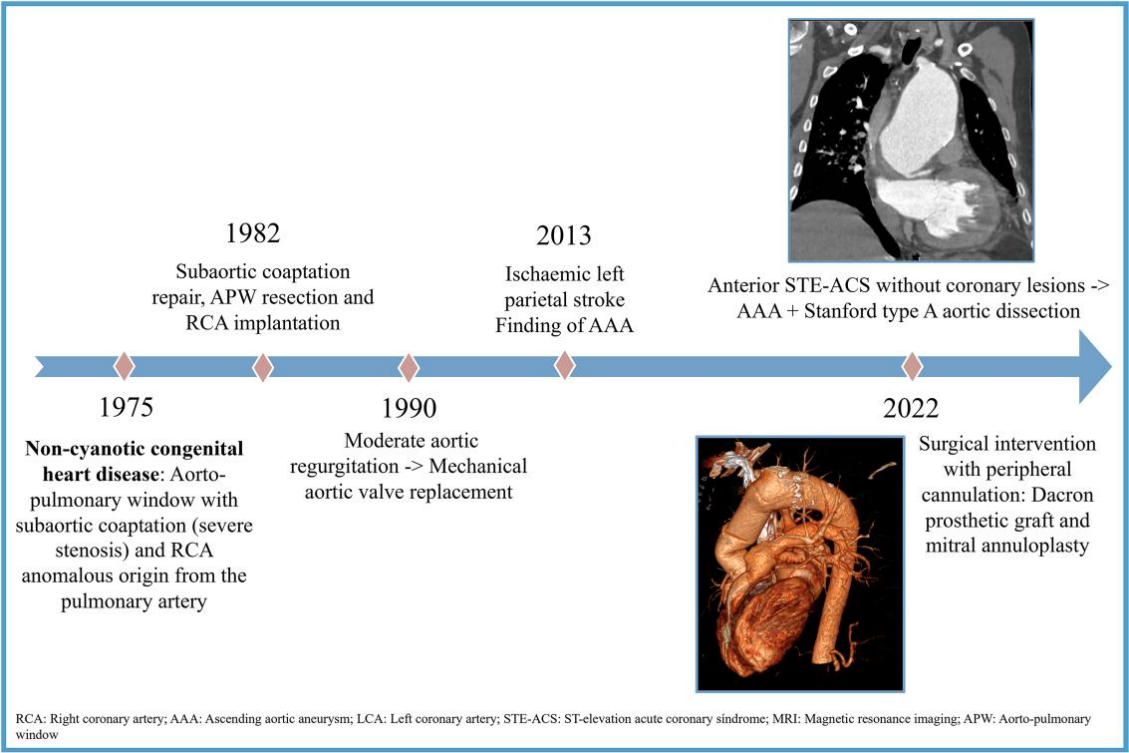


## Case presentation

A 47-year-old man with a childhood diagnosis of aortopulmonary window (APW) with severe subaortic stenosis and anomalous origin of the right coronary artery from the pulmonary artery (ARCAPA), who had undergone surgery at 8 years of age. The procedure involved resectioning the subaortic membrane, repairing the APW, and reimplanting the right coronary artery. At 15 years of age, he required aortic valve replacement with a mechanical prosthesis due to severe valve regurgitation. At 27, he was diagnosed with a 62-mm AAA. Despite recommendations for aortic surgery, the patient declined because of logistical reasons and returned to his home country. Antihypertensive medications were prescribed to manage his blood pressure and prevent any progression of the aneurysm or complications.

In October 2022, the patient presented to the emergency department with chest pain. He was hemodynamically stable (HR 90 bpm; BP 145/80 mmHg), cardiac auscultation with rhythmic tones and systolic murmur II/IV in mitral area and normal pulmonary auscultation; without ankle swelling. His electrocardiogram indicated anterior wall myocardial infarction with elevated troponin levels [553 ng/mL (normal value < 11 ng/mL)].

Echocardiography revealed moderate left ventricular systolic dysfunction with anterior and apical regional wall motion abnormalities, severe functional mitral regurgitation, and no pericardial effusion.

**Figure 1:**
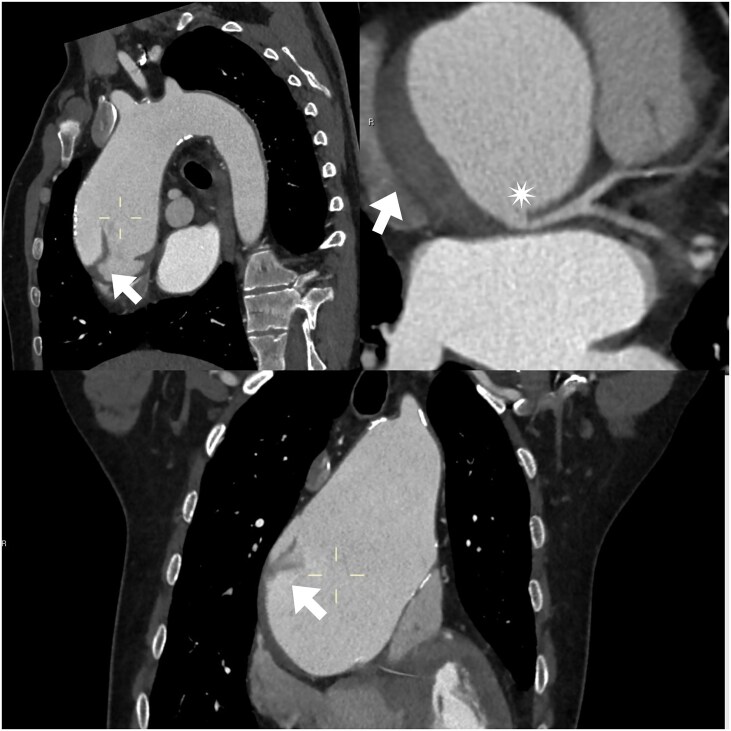
CT angiography image (sagittal, oblique and coronal). Giant ascending aortic aneurysm with Stanford type A dissection. It shows (arrows) the beginning and end of the dissection flap, without involvement of the LCA origin (star) or supra-aortic trunks.

Because of his background, an urgent chest CT angiography was performed; this revealed a large AAA (85 mm) with a Stanford type A dissection and permeability of both coronary ostia (*Figure 1*). It was decided not to administer antiplatelet therapy due to the absence of coronary lesions on aortic CT angiography.

**Figure 2:**
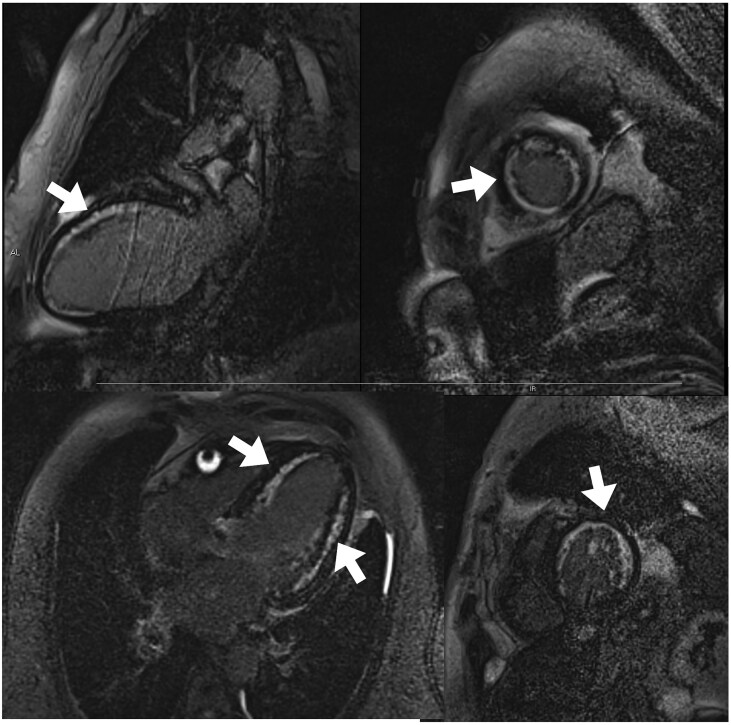
Cardiac MR image. Anterior and apical non-transmural late gadolinium enhancement sequences, 2 and 4-chamber view and basal and mid-short axis (indicated by arrows).

He was admitted to the coronary care unit for monitoring; electrical changes suggestive of infarction were transient, with normalisation of the ST segment. The case was discussed with cardiovascular surgeons, who agreed to delay surgery until the acute phase of the infarction had passed, given that the patient was hemodynamically stable. As part of the study, cardiac MRI was performed; this revealed akinesia in the region of the LCA and non-transmural late gadolinium enhancement (40%) (*Figure 2*). He was also diagnosed with severe functional mitral regurgitation.

**Figure 3:**
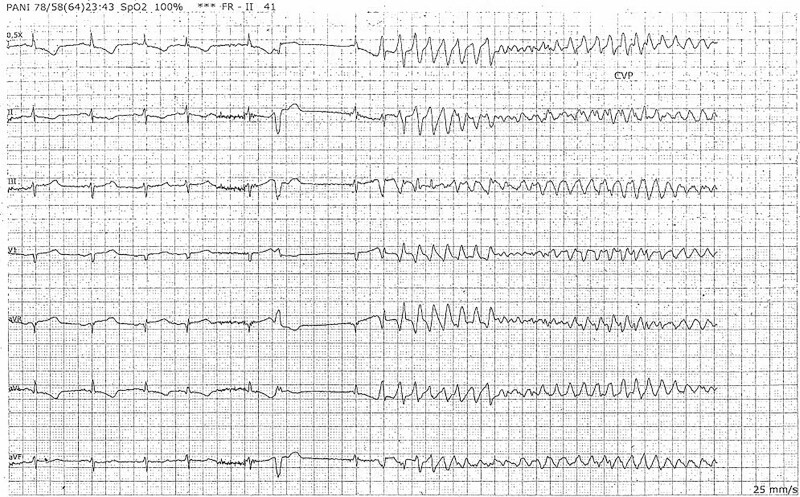
Telemetry record printout. Ventricular fibrillation.

Seventy-two hours after admission, the patient developed ventricular fibrillation as a result of a Valsalva maneuver associated with straining, requiring two electric shocks (*Figure 3*).

Once the patient's clinical condition had improved, surgery was planned. Mitral annuloplasty was performed with a 28 mm 4D memo ring, the coronary ostia were dissected, and a Dacron prosthetic graft was implanted in the ascending aorta with anastomosis to the aortic prosthesis and subsequent reinsertion of the coronary arteries.

**Figure 4:**
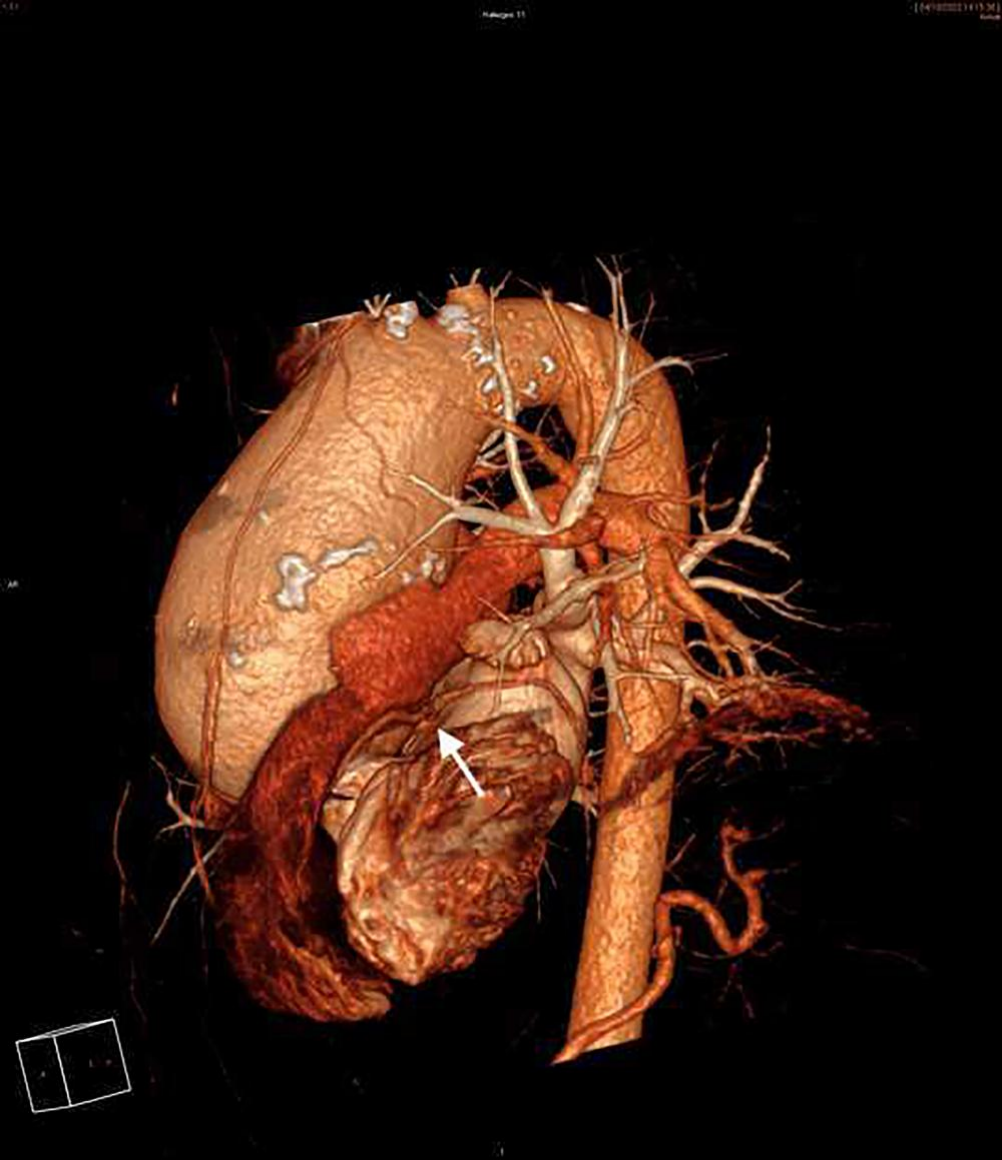
3D CT angiography reconstruction: giant aortic aneurysm and LCA (arrow).

**Figure 5:**
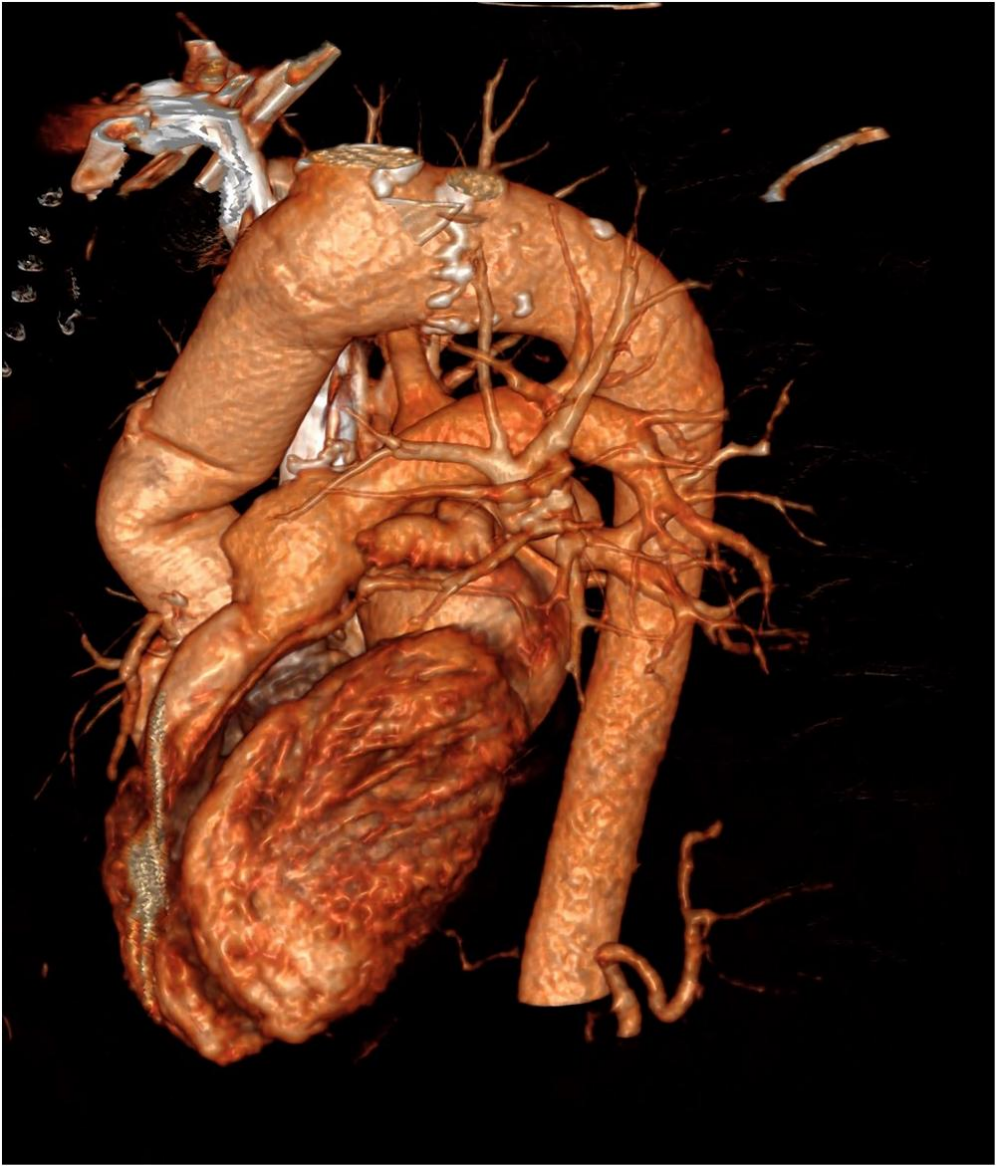
3D CT angiography reconstruction: post-surgical imaging.

The patient recovered well over the following days. The result of the surgery was verified by CT angiography (*Figures 4 and 5*), although echocardiography revealed the persistence of ventricular dysfunction.

During outpatient follow-up, heart failure drug therapy has been titrated with good clinical evolution, and echocardiographic evaluations have been performed, showing good prosthetic function.

## Discussion

APW is a very rare congenital heart disease involving communication between the aorta and pulmonary artery, sometimes associated with anomalous implantation of the coronary arteries, ARCAPA being the most common. It may present in childhood with angina, dyspnoea, and even sudden death, although corrective surgery generally produces good results.^1^ At other times, however, aortopathies may develop in adulthood.^2,3^

According to current guidelines on diseases of the aorta, the best treatment for this patient’s giant aneurysm, diagnosed at the age of 27, would have been surgery with Dacron prosthetic graft implantation to avoid the complications he subsequently experienced, but the patient refused. Imaging surveillance would have been strongly recommended. This should have been performed, ideally, with echocardiography every 6 months, and with MRI or CT angiography if the study were limited by the size of the aneurysm.^4^

Between 1% and 5% of type A aortic dissections present as acute myocardial infarction, increasing mortality and the risk of complications.^5^ The presence of ascending AD should be suspected in patients with a history of aortic aneurysm, poorly controlled hypertension with no other cardiovascular risk factors, and in young patients with a low probability of coronary atherosclerosis. The differential diagnosis of AD associated with myocardial infarction is essential to avoid inappropriate therapeutic approaches, such as the administration of anticoagulants or thrombolytics, especially in cases requiring emergent surgery.^6,7^ CT angiography plays a key role in these situations and D-dimer ≥750 ng/mL also appears to be helpful for diagnosis.^8^

According to the classification of coronary ostial lesions leading to malperfusion, by Neri *et al*. there are 3 groups: Type A, ostial dissection; type B, dissection with false coronary channel; and type C, circumferential detachment with invagination of the inner cylinder of the coronary artery.^9^ In our patient’s case, none of the previously described LCA ostium conditions were present. During the acute phase, the dissection caused an abrupt increase in the diameter of the false lumen and ascending aorta, which may have resulted in external compression of the LCA (between the pulmonary artery, left atrium, and giant AAA) as the most likely mechanism of malperfusion. This idea was supported by the CT angiography and the transient clinical signs of infarction. According to the new TEM classification, our patient’s AD can be classified as: type A, entry E1 and malperfusion M0, as no evidence of coronary dissection was found.^10^

The increase in intrathoracic pressure, combined with the increased cardiac output and arterial pressure during the first phase of the Valsalva maneuver, may have contributed to transient ischaemia through a similar mechanism, leading to ventricular fibrillation.

Delaying surgery was a difficult decision, but it was based on evidence suggesting that early intervention during the acute phase of infarction may increase mortality. Delaying surgery for at least 48 h, until the ischaemia had resolved, was deemed a safer option.^11,12^

When there is evidence of coronary obstruction by dissection, percutaneous intervention gives better results than bypass grafting.^13^ Since there was no evidence of coronary obstruction or dissection on the CT angiography, we did not consider direct coronary revascularization, and bypass grafting was unnecessary.

In our patient, there was no evidence of ischaemia in other organs due to the dissection, and the surgical approach was also challenging given the location of the aneurysm in the thorax, in close proximity to the sternum. It was therefore decided that the best option would be a well-planned elective surgery. Ultimately, this required the initiation of extracorporeal circulation via femoral and carotid access to prevent aortic rupture during an attempt at central cannulation.

## Lead author biography


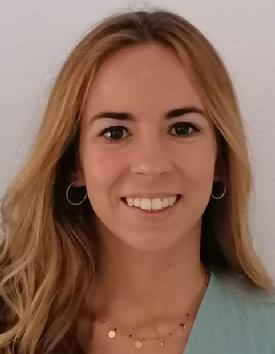


Cardiology Resident at the Hospital Virgen Macarena since 2020. PhD student at the University of Seville.

## Data Availability

Further echocardiography and CT images are available from the corresponding author.

## Notes

**Consent:** We have received the patient's informed consent for the use of data from their medical records and clinical images obtained for publication, in accordance with COPE guidelines.

**Funding:** None declared.
